# Supplementary material for: Genetic and Phenotypic Factors Affecting Glycemic Response to Metformin Therapy in Patients with Type 2 Diabetes Mellitus
Source: Genes (Basel). 2022 Jul 23;13(8):1310. doi: 10.3390/genes13081310 (PMC9330240; doi:10.3390/genes13081310)
Supplement: Supplementary file 1 [file genes-13-01310-s001.zip › genes-1820543-supplementary.pdf]

Supplementary Materials

Table S1. Primer sequences and PCR-RFLP details

Gene	SNP	Alleles	Primer sequence (5′→3′)	Restriction enzyme
ATM	rs11212617	C/A	F:CAGTAAAGTGAAGGAATACAGAGA	Bst4C I
			R:CTGTGTCTTTATATTTAAAGTGGG	
SLC2A2	rs8192675	A/G	F:TAATTTCAGGCCTGGTTCCTA	BstSF I
			R:AGATGGATGAAGTGGAGGAAG	
SLC22A1	rs628031	G/A	F:AGTCTCTGACTCATGCCTTTG	Mox20 I
			R:CCAGAGGCTTATCAAAGAGTC	
SLC22A1	rs12208357	C/T	F:CATCTGTGTGGGCATCGTCT	HspA I
			R:GTCATACACCCAGCCATCCT	
SLC47A1	rs2289669	G/A	F:CAGTTTCCACAGTAGCGTCG	Taq I
			R:CCCAGAGCGTTTCCTACCC	

Table S2. Genotype and allele frequencies of studied variants in patients with T2DM and control individuals

Genotype/allele	patients with T2DM n (%)	controls n (%)	p
**rs11212617** ***ATM***			
AA	136 (29)	41 (34)	0.3762
AC	228 (49)	53 (43)	0.3085
CC	100 (22)	28 (23)	07139
A	500 (54)	135 (55)	0.7183
C	428 (46)	109 (45)	
**rs628031** ***SLC22A1***			
AA	64 (14)	19 (16)	0.5577
AG	224 (48)	64 (52)	0.4763
GG	175 (38)	39 (32)	0.2469
A	352 (38)	102 (42)	0.3014
G	574 (62)	142 (58)	
**rs12208357** ***SLC22A1***			
CC	388 (84)	99 (80)	0.3451
CT	67 (15)	23 (19)	0.2617

TT	7 (2)	1 (1)	1.0000
C	843 (91)	221 (90)	0.5318
T	81 (9)	25 (10)	
**rs2289669 *SLC47A1***			
AA	83 (18)	19 (15)	0.4319
AG	149 (32)	37 (29)	0.5201
GG	232 (50)	73 (57)	0.1965
A	315 (34)	75 (29)	0.1547
G	613 (66)	183 (71)	
**rs8192675 *SLC2A2***			
AA	263 (57)	67 (55)	0.8369
AG	165 (36)	48 (40)	0.4580
GG	35 (8)	6 (5)	0.4244
A	691 (75)	182 (75)	0.9338
G	235 (25)	60 (25)	

Table S3. MAFs of studied variants in European populations

dbSNP ID	Gene	Allele	1000Genomes Project (European), %	GnomeAD Genome (Non-Finnish European), %	GnomeAD Exome (Non-Finnish European), %	Russian cohort, %
**rs11212617**	*ATM*	*A*	61.7	56.8	NA	43.7
**rs628031**	*SLC22A1*	*A*	41.4	40.7	41.7	41.5
**rs12208357**	*SLC22A1*	*T*	6.3	7.6	7.6	11,5
**rs2289669**	*SLC47A1*	*A*	41.6	41.0	NA	23.6
**rs8192675**	*SLC2A2*	*G*	28.9	29.4	NA	24.6

Table S4. Genotype and allele frequencies of studied variants in female and male patients with different glycemic response to metformin treatment

Genotype/allele	Female responders n (%)	Female non-responders n (%)	Male responders n (%)	Male non-responders n (%)	*p*-value
**rs11212617 *ATM***					
AA	51 (28)	15 (21)	5 (22)	6 (29)	p_1=0.6263 p_2=0.5540 p_3=0.2704 p_4=0.7322
AC	99 (54)	34 (47)	14 (61)	11 (52)	p_1=0.6581 p_2=0.8050 p_3=0.3333 p_4=0.7613
CC	33 (18)	23 (32)	4 (17)	4 (19)	p_1=1.0000 p_2=0.2897 **p_3=0.0190** p_4=1.0000
A	201 (55)	64 (44)	24 (52)	23 (55)	p_1=0.7550 p_2=0.2922 **p_3=0.0386** p_4=0.8336
C	165 (45)	80 (56)	22 (48)	19 (45)	
**rs628031 *SLC22A1***					
AA	19 (10)	9 (12)	3 (13)	5 (24)	p_1=0.7198 p_2=0.2951 p_3=0.6592 p_4=0.4485
AG	93 (51)	35 (49)	14 (61)	8 (38)	p_1=0.5071 p_2=0.4614 p_3=0.7812 p_4=0.2271
GG	70 (39)	28 (39)	6 (26)	8 (38)	p_1=0.3596 p_2=1.0000 p_3=1.0000 p_4=0.5206
A	131 (36)	53 (37)	20 (43)	18 (43)	p_1=0.3338 p_2=0.4771 p_3=0.9185 p_4=1.0000
G	233 (64)	91 (63)	26 (57)	24 (57)	
**rs12208357 *SLC22A1***					

CC	158 (86)	61 (87)	17 (74)	15 (71)	p_1=0.1260<br>p_2=0.1028<br>p_3=1.0000<br>p_4=1.0000
CT	23 (13)	9 (13)	4 (17)	5 (24)	p_1=0.5141<br>p_2=0.2992<br>p_3=1.0000<br>p_4=0.7159
TT	2 (1)	0 (0)	2 (9)	1 (5)	p_1=0.0624<br>p_2=0.2308<br>p_3=1.0000<br>p_4=1.0000
C	339 (93)	131 (94)	38 (83)	35 (83)	**p_1=0.0425**<br>p_2=0.0585<br>p_3=0.8474
T	27 (7)	9 (6)	8 (17)	7 (17)	p_4=1.0000
			**rs2289669 *SLC47A1***		
AA	24 (13)	13 (18)	7 (30)	4 (19)	p_1=0.0557<br>p_2=1.0000<br>p_3=0.3271<br>p_4=0.4941
AG	63 (34)	25 (35)	6 (26)	4 (19)	p_1=0.4900<br>p_2=0.1947<br>p_3=1.0000<br>p_4=0.7240
GG	96 (53)	34 (47)	10 (44)	13 (62)	p_1=0.5085<br>p_2=0.3236<br>p_3=0.4882<br>p_4=0.2457
A	111 (30)	51 (35)	20 (43)	12 (29)	p_1=0.0920<br>p_2=0.4625<br>p_3=0.2911
G	255 (70)	93 (65)	26 (57)	30 (71)	p_4=0.1851
			**rs8192675 *SLC2A2***		
AA	106 (58)	41 (57)	12 (52)	8 (38)	p_1=0.6564<br>p_2=0.1441<br>p_3=0.8883<br>p_4=0.3815
AG	62 (34)	28 (39)	9 (39)	12 (57)	p_1=0.6468<br>p_2=0.2098<br>p_3=0.4709<br>p_4=0.3651
GG	14 (8)	3 (4)	2 (9)	1 (5)	p_1=0.6964<br>p_2=1.0000<br>p_3=0.4102<br>p_4=1.0000

A	274 (75)	110 (76)	33 (72)	28 (67)	p_1=0.5917<br>p_2=0.2310<br>p_3=0.8198<br>p_4=0.6490
G	90 (25)	34 (24)	13 (28)	14 (33)	

P-value ≤ 0.05 was considered statistically significant and is shown in bold; p_1: *p*-value calculated for female responders compared to male responders; p_2: p-value calculated for female non-responders compared to male non-responders; p_3: *p*-value calculated for female responders compared to female non-responders; p_4: *p*-value calculated for male responders compared to male non-responders.

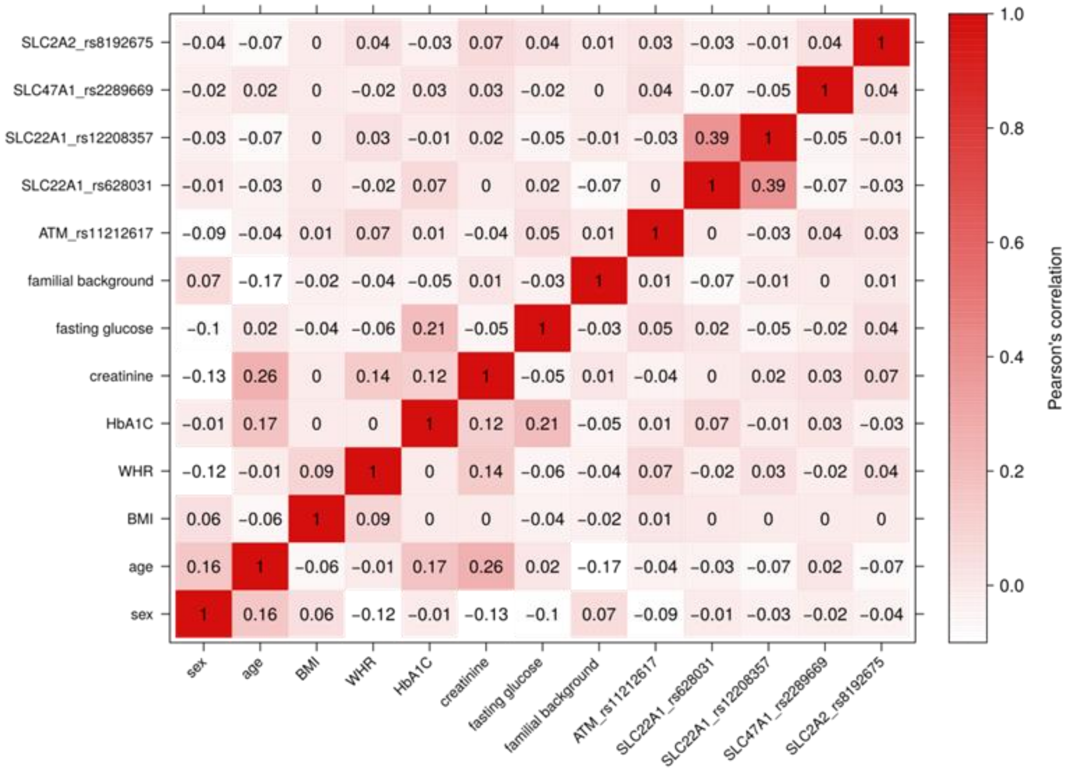

Figure S1. A heatmap showing Pearson's correlation coefficient between variables used to fit predictive models of response to metformin therapy. For SNPs, the value was calculated using the vectors of non-reference allele counts. Note that no pair of variables correlate with r2 > 0.6, a commonly used threshold for exclusion of predictors.

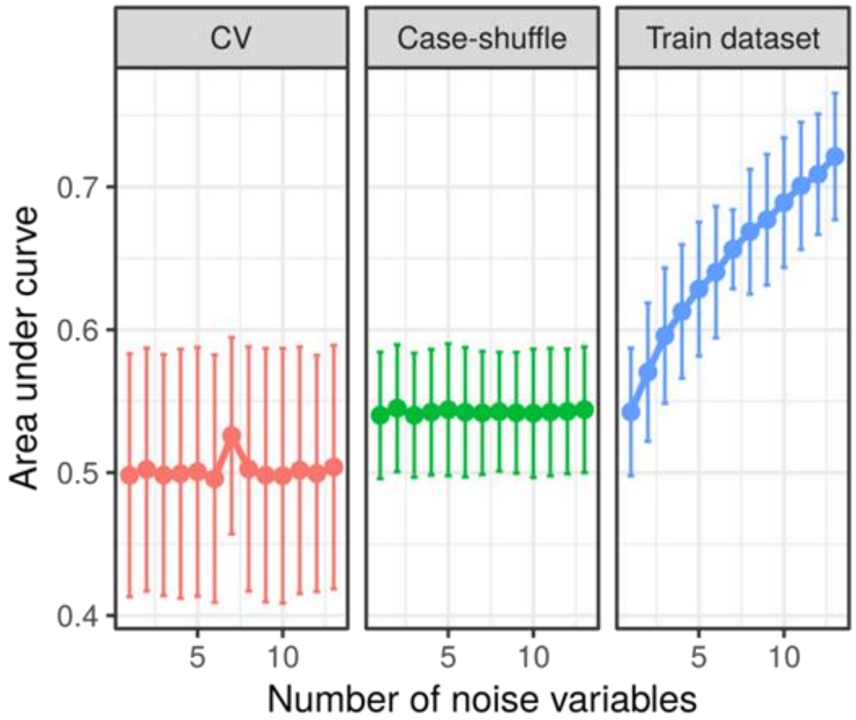

Figure S2. Baseline efficiency of predicting response to metformin based on the different number of random noise variables used as predictors. Mean and standard deviation of the area under curve (AUC) values calculated using 4-fold cross-validation (CV), case-shuffle (see Methods), or using the complete training dataset.

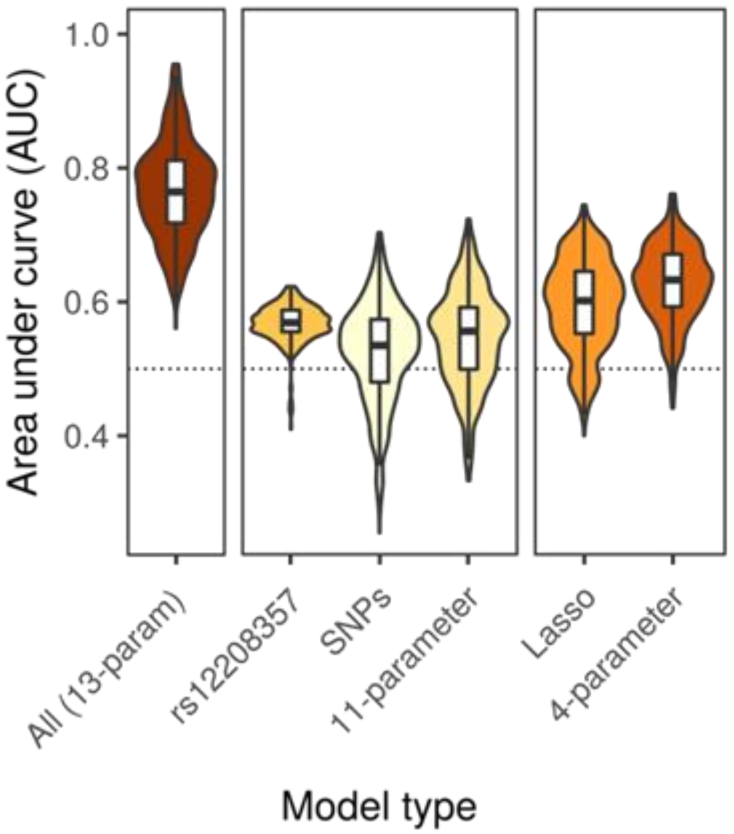

Figure S3. Cross validation-based estimates of the efficiency of predicting response to metformin treatment using genotypes and additional phenotypic features. Area under receiver-operator (ROC) curve (AUC) values were estimated using a 4-fold cross-validation strategy for model evaluation. Scores are shown for different types of models (see main text). Dashed line indicates an AUC = 0.5 threshold corresponding to the performance of random noise classification in the same 4-fold cross-validation test. Note that the estimated performance of all models is lower than in the "case-shuffle" strategy used in Figure 1a. The differences reflect the levels of bias in different model evaluation strategies (Figure S2).

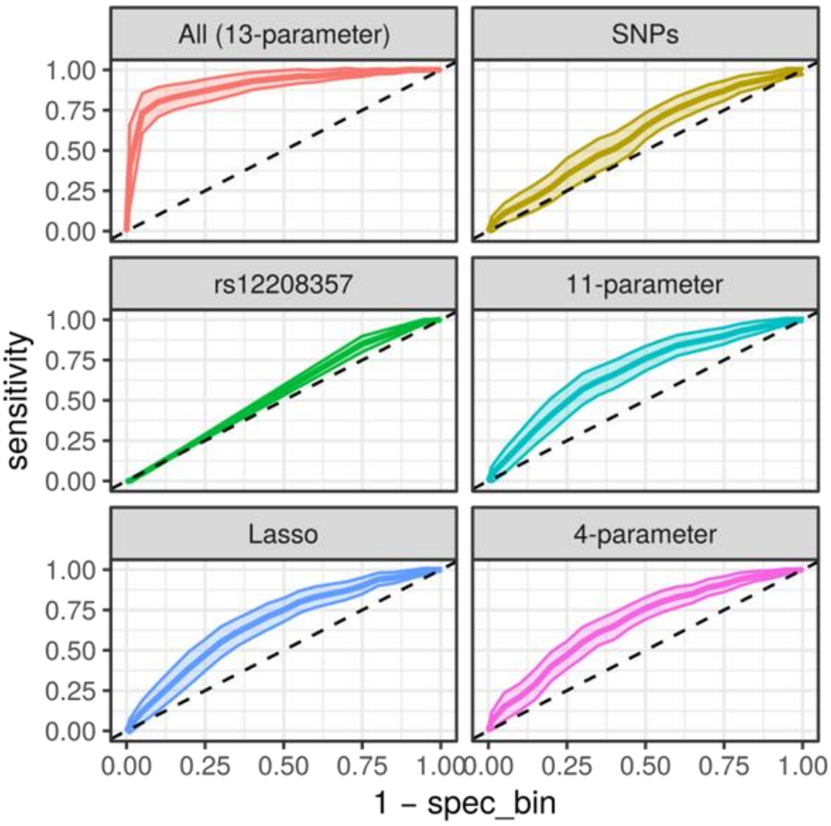

Figure S4. Receiver-operator (ROC) curves for different types of models for prediction of response to metformin treatment using genotypes and additional phenotypic features.
